# Two-period demand phenotypes of oliceridine-based patient-controlled intravenous analgesia after gynecologic surgery: perioperative correlates of persistent high demand

**DOI:** 10.3389/fmed.2026.1970542

**Published:** 2026-10-14

**Authors:** Dandan Chen, Ting Qiu, Xiaoquan Yu, Lu Wang, Yuqian Chen

**Affiliations:** 1 Department of Anesthesiology, Quanzhou First Hospital Affiliated to Fujian Medical University, Quanzhou, China; 2 Department of Anesthesiology, Longyan First Hospital Affiliated to Fujian Medical University, Longyan, China

**Keywords:** analgesic demand phenotype, dexmedetomidine, gynecologic surgery, multimodal analgesia, oliceridine, patient-controlled intravenous analgesia, postoperative opioid consumption, regional analgesia

## Abstract

**Background:**

Postoperative opioid requirements vary substantially despite standardized analgesic pathways, and cumulative consumption may obscure temporal heterogeneity. We characterized two-period demand phenotypes of oliceridine-based patient-controlled intravenous analgesia (PCIA) after gynecologic surgery and examined perioperative correlates of consumption.

**Methods:**

This single-center retrospective cohort included adult women who underwent elective gynecologic surgery and received oliceridine-based PCIA in 2025. The protocol-specified primary outcome was cumulative 48-h oliceridine consumption. Multivariable linear regression with heteroscedasticity-consistent type 3 (HC3) robust standard errors evaluated its perioperative correlates. Exploratory *post hoc* phenotypes were defined from cumulative 0–24 h and incremental 24–48 h consumption using cohort-specific third-quartile thresholds (19.6 and 18.2 mg): low/intermediate, early high, late-increasing, and persistent high demand. Phenotype-specific and high 48-h consumption (>38.1 mg) logistic models and sensitivity analyses assessed consistency.

**Results:**

Among 325 patients, 224 (68.9%) had low/intermediate demand, 20 (6.2%) early high demand, 20 (6.2%) late-increasing demand, and 61 (18.8%) persistent high demand. In the primary continuous-outcome model (*n* = 319), each 10-year increase in age was associated with 2.73 mg lower 48-h consumption, whereas each 60-min increase in surgical duration, open surgery, and hysterectomy were associated with 2.73, 6.85, and 5.26 mg higher consumption, respectively. Regional block and intraoperative dexmedetomidine were each associated with reductions of 2.58 and 3.25 mg, respectively. In the exploratory phenotype model, younger age, longer surgery, open surgery, and hysterectomy were associated with persistent high demand, whereas regional block and intraoperative dexmedetomidine were each inversely associated. Compared with neither intervention, the combination of regional block and intraoperative dexmedetomidine was associated with lower odds of persistent high demand (adjusted odds ratio, 0.17; 95% confidence interval, 0.05–0.50) and an adjusted 48-h consumption that was 5.9 mg lower.

**Conclusion:**

Perioperative correlates of cumulative 48-h oliceridine consumption were broadly concordant with those identified using the exploratory two-period phenotype framework. These findings may support risk-stratified acute pain surveillance, but they require prospective external validation.

**Study registration:**

Retrospectively registered with the Chinese Clinical Trial Registry (ChiCTR2600128515) after completion of data collection.

## 1 Introduction

Despite standardized perioperative pathways and the widespread use of multimodal analgesia, acute postoperative pain and opioid requirements vary substantially among patients. Previous systematic and prospective studies have associated this heterogeneity with age, preoperative pain and psychological characteristics, procedure type, surgical approach, and other perioperative factors (1–3). In women undergoing hysterectomy, younger age and an open surgical approach have also been associated with greater postoperative opioid consumption (2, 3). These findings support individualized postoperative analgesic surveillance rather than reliance on a uniform analgesic regimen (4–6).

Oliceridine is an intravenous *μ*-opioid receptor agonist with preferential G-protein signaling (7), developed for the treatment of moderate-to-severe acute pain when intravenous opioid therapy is warranted. The phase III APOLLO-1 and APOLLO-2 trials established its analgesic efficacy in patients undergoing bunionectomy and abdominoplasty, respectively (8, 9). More recent randomized studies in laparoscopic total hysterectomy and gynecologic laparoscopic surgery have compared oliceridine with morphine or sufentanil, focusing primarily on analgesic efficacy, postoperative nausea and vomiting, respiratory-related events, and quality of recovery (10, 11). Beyond these controlled comparisons, the phase III ATHENA study evaluated oliceridine in a broader multicenter clinical population, in which the drug was administered by clinician-directed bolus and/or patient-controlled analgesia as part of multimodal analgesic care, while a subsequent meta-analysis synthesized randomized evidence across postoperative pain models (12, 13). However, these studies principally addressed treatment efficacy and safety. Evidence remains limited regarding within-cohort variability in actual oliceridine-based patient-controlled intravenous analgesia (PCIA) consumption and how demand evolves during the first 48 postoperative hours.

Postoperative analgesic consumption is commonly summarized as a single cumulative dose over a predefined interval. However, prospective cohort studies have demonstrated that postoperative pain and opioid consumption may follow distinct temporal trajectories across surgical populations (14–16). Consequently, a 48-h cumulative dose may identify overall high consumption but cannot distinguish patients with predominantly early demand from those whose requirements increase or remain high during the second postoperative day. When PCIA records provide cumulative consumption at 24 and 48 h but not sufficiently granular event-level data for formal trajectory modeling, separating 0–24 h cumulative consumption from the 24–48 h incremental dose may offer a pragmatic approach to describing temporal heterogeneity in analgesic demand.

This study therefore aimed to characterize rule-based two-period phenotypes of oliceridine-based PCIA demand using 0–24 h cumulative consumption and 24–48 h incremental consumption in women undergoing gynecologic surgery under a relatively standardized anesthetic pathway. We further evaluated patient- and surgery-related correlates of persistent high demand and examined whether modifiable perioperative analgesic exposures, particularly regional block and intraoperative dexmedetomidine, individually and in combination, were associated with lower oliceridine requirements. We hypothesized that greater surgical burden would be associated with persistent high demand, whereas multimodal analgesic exposures would be associated with lower demand.

## 2 Materials and methods

### 2.1 Study design, setting, and ethics

This single-center retrospective cohort study was conducted at Quanzhou First Hospital between January 1 and December 31, 2025. Data were retrieved from routinely collected electronic medical records, anesthesia information system records, nursing documentation, acute pain service (APS) follow-up records, and patient-controlled intravenous analgesia (PCIA) device records. No additional interventions, examinations, medications, or follow-up procedures were performed for research purposes.

The study was approved by the Ethics Committee of Quanzhou First Hospital on April 14, 2026 (approval no. 泉一伦〔2026〕K150). The requirement for written informed consent was waived by the Ethics Committee because of the retrospective design, the use of de-identified routinely collected data, and the minimal risk to participants. The study was conducted in accordance with the Declaration of Helsinki and relevant institutional regulations and was reported in accordance with the Strengthening the Reporting of Observational Studies in Epidemiology (STROBE) statement (17). The study was retrospectively registered with the Chinese Clinical Trial Registry (ChiCTR2600128515) after completion of data collection.

### 2.2 Study population

All consecutive adult women who underwent elective gynecologic surgery during the study period were screened for eligibility. Patients were included if they met all of the following criteria: age ≥18 years; elective gynecologic surgery, including hysterectomy, adnexal surgery, myomectomy, radical gynecologic oncologic surgery, or another gynecologic procedure; American Society of Anesthesiologists (ASA) physical status I–III; postoperative use of oliceridine-based PCIA; and valid cumulative oliceridine consumption records for both the 0–24 h and 0–48 h postoperative observation windows.

Patients were excluded if they had pre-existing chronic pain, long-term regular opioid use, or documented opioid tolerance; severe psychiatric disease or cognitive impairment that could compromise pain assessment; concomitant use of another continuous opioid analgesic regimen that precluded reliable assessment of oliceridine consumption; missing key clinical or primary outcome data; reoperation within 48 h after surgery; or a major postoperative complication that substantially altered postoperative analgesic requirements.

Early PCIA discontinuation did not in itself result in exclusion. Patients with early discontinuation were retained in the final cohort when the actual cumulative oliceridine dose administered during the 0–24 h and 0–48 h observation windows could be reliably ascertained from PCIA device records and APS documentation. Patients who used PCIA for <48 h but lacked valid cumulative 48-h consumption data were excluded. The potential influence of early discontinuation was evaluated in sensitivity analyses that excluded these patients.

### 2.3 Anesthetic and postoperative analgesic management

All patients received general anesthesia with endotracheal intubation under a standardized institutional protocol, with individualized titration as clinically indicated. Anesthesia was induced with intravenous sufentanil 0.5 μg/kg, propofol 2 mg/kg, and cisatracurium 0.2 mg/kg. Anesthesia was maintained using target-controlled infusions of propofol at a target concentration of approximately 3 mg/L and remifentanil at approximately 1–2 μg/L. Target concentrations were individualized within these institutional ranges and adjusted according to the intensity of surgical stimulation, depth of anesthesia, and the patient's hemodynamic response. Additional cisatracurium was administered when clinically indicated. Dolasetron 12.5 mg was administered intravenously before the end of surgery for prophylaxis against postoperative nausea and vomiting.

The use of intraoperative dexmedetomidine, non-steroidal anti-inflammatory drugs (*N*SAIDs), regional block, and wound infiltration with local anesthetic was determined by the attending anesthesiologist according to the patient's clinical condition, surgical procedure, and routine institutional practice. These interventions were part of routine clinical care, were not assigned for research purposes, and were recorded as perioperative analgesic exposures.

During the study period, regional blocks were generally ultrasound-guided and predominantly consisted of transvs. abdominis plane blocks. Ropivacaine 0.25% or 0.375% was typically administered at approximately 20 mL per side without routine addition of dexamethasone or dexmedetomidine. Wound infiltration generally used approximately 20 mL of 0.5% ropivacaine. These descriptions reflect prevailing institutional practice rather than patient-level exposure data.

Intraoperative dexmedetomidine use was at the discretion of the attending anesthesiologist and was titrated to the patient's hemodynamic status and clinical requirements. The analytic dataset did not consistently retain the exact loading dose, maintenance infusion rate, initiation time, or duration; dose-response analyses were therefore not feasible.

At the end of surgery, residual neuromuscular blockade was reversed when clinically indicated. Tracheal extubation was performed after recovery of consciousness, adequate spontaneous ventilation, and airway protective reflexes. Patients were subsequently transferred to the post-anesthesia care unit and returned to the ward after meeting routine discharge criteria.

The postoperative PCIA solution contained oliceridine 50 mg and dolasetron 25 mg diluted with 0.9% sodium chloride to a total volume of 100 mL. This admixture reflected routine institutional practice; however, its physicochemical compatibility and stability were not formally evaluated in the present retrospective study. The basal infusion rate was 0.5 mL/h, corresponding to an oliceridine infusion rate of 0.25 mg/h. The patient-controlled bolus dose was 4 mL, corresponding to 2 mg of oliceridine, with a lockout interval of 10 min. The planned duration of PCIA treatment was 48 h.

Postoperative analgesic management followed the routine institutional APS pathway, with assessments on postoperative days 1 and 2. Rescue analgesia could include intravenous sufentanil, oxycodone, or flurbiprofen axetil; agent and dose were individualized according to pain severity, prior response, and clinical condition rather than a fixed protocol. For patients whose PCIA settings were adjusted, consumption analyses used the actual cumulative oliceridine dose recorded by the device.

The intraoperative opioid regimen was protocolized, including weight-based sufentanil administration during induction and remifentanil target-controlled infusion within a predefined institutional target range. However, complete pump-derived cumulative remifentanil doses and event-level infusion histories were not consistently retrievable from the archived anesthesia records for the entire cohort. Because remifentanil was administered using target-controlled infusion, cumulative exposure could not be reliably reconstructed from the available target-setting information. Quantitative intraoperative remifentanil exposure was therefore not included as a separate covariate in the multivariable analyses.

### 2.4 Data collection and variable definitions

Data were extracted according to a prespecified data dictionary. Demographic characteristics and comorbidities were obtained from the electronic medical record system; surgical and anesthetic variables were obtained from the anesthesia information system; postoperative pain scores, APS interventions, and adverse events were obtained from nursing and APS documentation; and oliceridine consumption and PCIA demand counts were obtained from PCIA device records.

Demographic and preoperative variables included age, height, weight, body mass index (BMI), American Society of Anesthesiologists (ASA) physical status, smoking history, hypertension, diabetes mellitus, and history of postoperative nausea and vomiting (PONV).

Disease- and surgery-related variables included benign or malignant disease, surgical type, surgical approach, hysterectomy, adnexectomy, lymph node dissection, surgical duration, estimated blood loss, and perioperative blood transfusion. Surgical type was categorized as adnexal surgery, hysterectomy, myomectomy, radical gynecologic oncologic surgery, or other gynecologic surgery. Surgical approach was categorized as open surgery or laparoscopy. Open surgery was defined as surgery performed through laparotomy, whereas minimally invasive procedures were categorized as laparoscopy. Surgical duration was defined as the interval from skin incision to completion of wound closure, as recorded in the anesthesia information system.

Perioperative analgesic exposures included intraoperative NSAIDs, dexmedetomidine, regional block, and wound infiltration. Dexmedetomidine, regional block, and wound infiltration were recorded as dichotomous exposures. Patient-level details on dexmedetomidine dose and timing, block type and local-anesthetic regimen, block adjuvants, and wound-infiltration regimen were not consistently available. For an exploratory *post hoc* combination analysis, regional block and intraoperative dexmedetomidine were additionally represented as a four-level exposure variable: neither intervention, dexmedetomidine only, regional block only, or both interventions.

PCIA-related variables included cumulative oliceridine consumption at 24 and 48 h; device-recorded effective PCIA demands within 48 h, defined as demands resulting in bolus delivery; total PCIA demands within 48 h; PCIA parameter adjustment; early PCIA discontinuation; and the documented reason for early discontinuation, when applicable.

Postoperative outcomes included resting and movement numerical rating scale (NRS) pain scores on postoperative days 1 and 2, PONV within 48 h, rescue analgesia within 48 h, the number of rescue-analgesia episodes, dizziness, somnolence or sedation, pruritus, respiratory depression, and postoperative length of stay.

Movement NRS was defined as the maximum pain score recorded during turning, coughing, or ambulation within the corresponding postoperative assessment period. PONV was defined as nausea, vomiting, or the requirement for additional antiemetic therapy within 48 h after surgery. Rescue analgesia was defined as any additional analgesic intervention administered within 48 h because of insufficient pain control. Commonly used rescue agents in routine practice included sufentanil, oxycodone, and flurbiprofen axetil. The specific agent and dose were individualized according to clinical need, and detailed patient-level dose information was not consistently available in the analytic dataset. Adverse events were identified from nursing and APS documentation recorded during the first 48 postoperative hours.

### 2.5 Oliceridine consumption outcomes

The protocol-specified primary outcome was the actual cumulative dose of oliceridine administered during the first 48 postoperative hours, measured in milligrams and obtained from PCIA device records. Cumulative 0–24 h oliceridine consumption was defined as the device-recorded cumulative dose at 24 h.

Incremental oliceridine consumption during the subsequent postoperative period was calculated for each patient as:

24–48 h incremental consumption = cumulative 48-h consumption−cumulative 24-h consumption.

The protocol-specified binary high-consumption outcome was defined as cumulative 48-h oliceridine consumption above the third quartile of the overall cohort. The observed third-quartile value was 38.1 mg; therefore, high 48-h consumption was defined using a strict threshold of >38.1 mg.

### 2.6 Exploratory two-period demand phenotype classification

To characterize temporal heterogeneity in postoperative oliceridine demand, an exploratory, rule-based two-period classification was developed using cumulative consumption during 0–24 h and incremental consumption during 24–48 h. Because oliceridine consumption was available at only two assessment times, formal longitudinal trajectory modeling was not performed.

Third-quartile thresholds were calculated in the overall cohort using the type 7 sample-quantile estimator in R version 4.6.0 (R Foundation for Statistical Computing, Vienna, Austria). The resulting Q3 thresholds were 19.6 mg for cumulative 0–24 h consumption and 18.2 mg for incremental 24–48 h consumption. These cohort-derived thresholds were used solely to construct exploratory descriptive phenotypes and were not intended to represent externally validated clinical cut points. High early demand and high late-period demand were defined using strict > Q3 thresholds; values equal to the respective Q3 thresholds were classified as not high.

Patients were assigned to one of four mutually exclusive demand phenotypes:

Low/intermediate demand: 0–24 h consumption ≤19.6 mg and 24–48 h incremental consumption ≤18.2 mg;
Early high demand: 0–24 h consumption >19.6 mg and 24–48 h incremental consumption ≤18.2 mg;
Late-increasing demand: 0–24 h consumption ≤19.6 mg and 24–48 h incremental consumption >18.2 mg; and
Persistent high demand: 0–24 h consumption >19.6 mg and 24–48 h incremental consumption >18.2 mg.

The persistent high-demand phenotype served as the outcome in an exploratory *post hoc* phenotype-specific multivariable model. The protocol-specified high 48-h consumption outcome (>38.1 mg) was examined in a secondary model to assess whether the direction and approximate magnitude of perioperative associations were consistent with those for the exploratory phenotype outcome.

### 2.7 Statistical analysis

Continuous variables were assessed for distributional characteristics and summarized as median and interquartile range (IQR), whereas categorical variables were summarized as number and percentage. Comparisons across the four demand-phenotype groups were performed using the Kruskal–Wallis test for continuous variables. Categorical variables were compared using Pearson's chi-squared test when expected-frequency assumptions were satisfied. Fisher's exact test was used for sparse 2 × 2 tables, and Fisher's exact test with Monte Carlo simulation using 1,000,000 replicates was used for larger sparse contingency tables. Because the phenotype-group comparisons were primarily descriptive and exploratory, no *post hoc* pairwise comparisons were conducted. No formal sample-size calculation was performed because all eligible patients within the prespecified retrospective study period were included; for the exploratory phenotype-specific analysis, model complexity was constrained by the number of persistent high-demand events and the clinical relevance of the candidate covariates.

For analysis of the protocol-specified primary continuous outcome, cumulative 48-h oliceridine consumption was modeled using multivariable linear regression with heteroscedasticity-consistent type 3 (HC3) robust standard errors. The model included six clinically selected preoperative or intraoperative covariates: age, surgical duration, open surgery versus laparoscopy, hysterectomy performed, regional block, and intraoperative dexmedetomidine. These covariates were selected based on clinical relevance, biological plausibility, data availability, and the number of outcome events; no automated stepwise selection procedure was used. Age was modeled per 10-year increase and surgical duration per 60-min increase. Regression coefficients were reported as adjusted mean differences in cumulative 48-h oliceridine consumption, expressed in milligrams, with 95% confidence intervals (CIs).

An exploratory *post hoc* phenotype-specific analysis evaluated factors associated with persistent high demand versus all other phenotypes. A multivariable logistic regression model included the same six covariates as the protocol-specified continuous-outcome model, and associations were reported as adjusted odds ratios (ORs) with 95% CIs. With 57 persistent high-demand events and six prespecified covariates, the model had approximately 9.5 events per covariate. Model complexity was therefore deliberately restricted, without additional data-driven variable selection. A secondary logistic regression model used the protocol-specified high 48-h consumption outcome (>38.1 mg) and included the same covariates.

To examine clinically modifiable analgesic exposures, patients were classified into four mutually exclusive groups according to receipt of regional block and intraoperative dexmedetomidine: neither intervention, dexmedetomidine only, regional block only, or both interventions. The group receiving neither intervention served as the reference category. Associations with persistent high demand were estimated using multivariable logistic regression adjusted for age, surgical duration, open surgery, and hysterectomy. A parallel linear regression model with heteroscedasticity-consistent type 3 (HC3) robust standard errors evaluated cumulative 48-h oliceridine consumption. Adjusted marginal probabilities of persistent high demand and adjusted marginal mean consumption were estimated by standardizing model predictions over the observed covariate distribution.

Postoperative pain scores, postoperative nausea and vomiting, rescue analgesia, adverse events, PCIA parameter adjustment, and early PCIA discontinuation were treated as postoperative outcomes or exploratory correlates and were not included in the prespecified multivariable models. These variables were measured during the same 48-h period as the consumption outcomes and could represent consequences, intermediate variables, or clinical responses to analgesic demand. Multivariable analyses used complete cases; no imputation was performed because missingness was <5% for all model covariates.

Sensitivity analyses sequentially excluded patients with: (1) any early PCIA discontinuation; (2) non-depletion-related early discontinuation due to adverse events, patient refusal, or a change in analgesic strategy; (3) unexplained low-consumption records, defined as cumulative 48-h consumption <12 mg without documented early discontinuation or PCIA parameter adjustment; (4) cumulative 48-h consumption of 49 mg, indicating consumption near the 50-mg reservoir ceiling; or (5) PCIA parameter adjustment. A strict sensitivity set excluded patients with either non-depletion-related early discontinuation or unexplained low-consumption records. Phenotype thresholds derived from the overall cohort were retained across all sensitivity analyses to avoid outcome reclassification caused solely by changes in the analysis sample.

All tests were two-sided, with *P* < 0.05 considered statistically significant. Because coefficients in the clinically selected multivariable models were estimated simultaneously, they were not treated as a family of *post hoc* pairwise comparisons. In the exploratory four-group regional block/dexmedetomidine analysis, Holm adjustment was applied to the three contrasts with the neither-intervention reference group separately for persistent high demand and cumulative 48-h consumption. Effect estimates and 95% CIs were emphasized; nominal *P* values between 0.05 and 0.10 were considered directionally suggestive rather than statistically significant.

## 3 Results

### 3.1 Cohort selection and phenotype distribution

A total of 368 patient records were screened for eligibility. Forty-three records were excluded because of chronic pain or long-term opioid use (*n* = 4), PCIA use for <48 h without valid cumulative 48-h consumption data or incomplete 48-h consumption records (*n* = 19), missing key data (*n* = 12), reoperation or major complications within 48 h (*n* = 3), or use of another continuous opioid analgesic regimen (*n* = 5). The final cohort comprised 325 patients (Figure 1).

**Figure 1:**
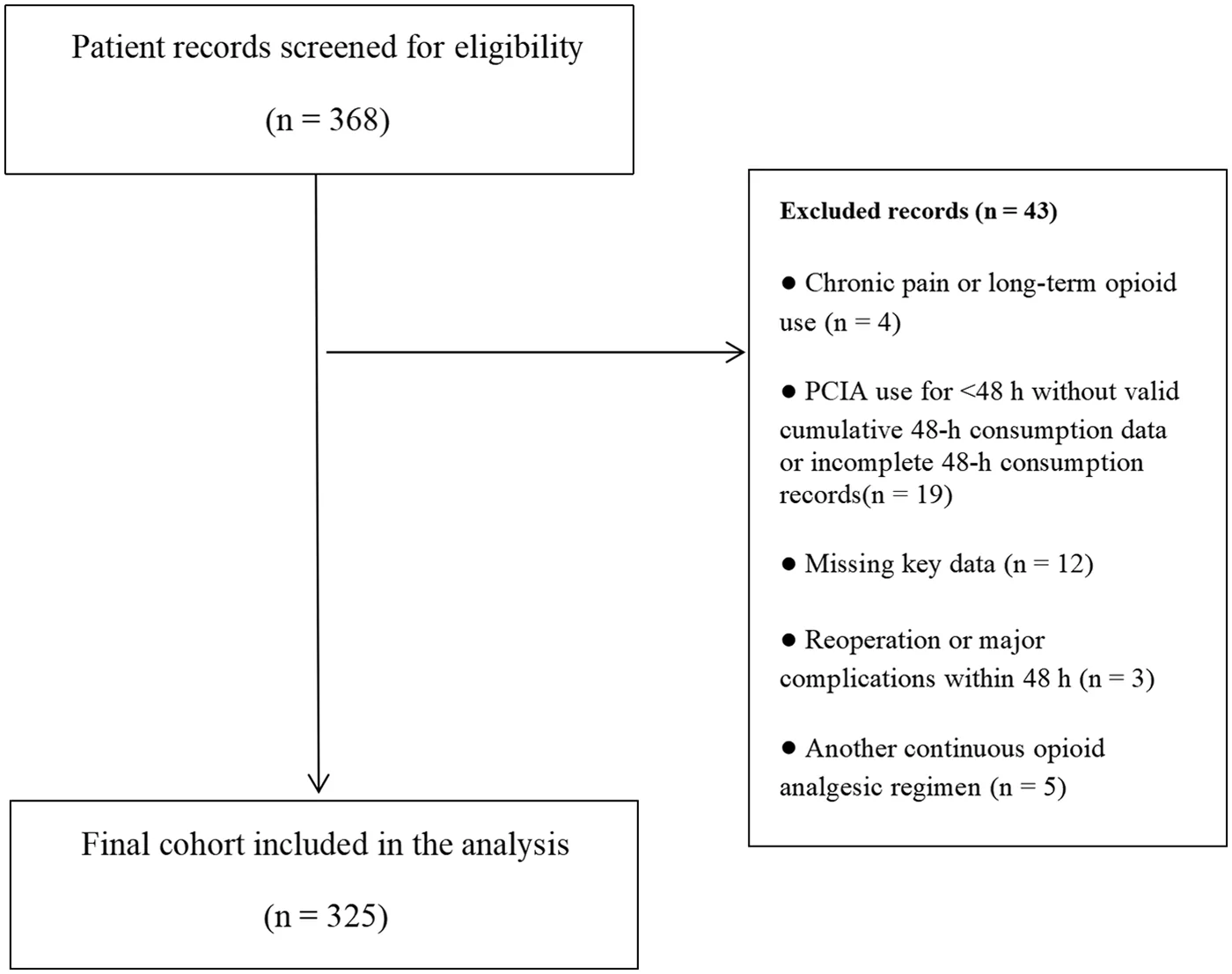
Patient selection flow diagram. A total of 368 patient records were screened for eligibility. Forty-three records were excluded because of chronic pain or long-term opioid use (*n* = 4), PCIA use for <48 h without valid cumulative 48-h consumption data or incomplete 48-h consumption records (*n* = 19), missing key data (*n* = 12), reoperation or major complications within 48 h (*n* = 3), or use of another continuous opioid analgesic regimen (*n* = 5). The final cohort comprised 325 patients. PCIA, patient-controlled intravenous analgesia.

Median cumulative oliceridine consumption was 15.4 mg (IQR, 11.5–19.6) during 0–24 h and 30.1 mg (IQR, 22.7–38.1) during 0–48 h. In the overall cohort, the third-quartile thresholds were 19.6 mg for 0–24 h cumulative consumption, 18.2 mg for 24–48 h incremental consumption, and 38.1 mg for cumulative 48-h consumption.

Based on the early and late-period thresholds, 224 patients (68.9%) were classified as having low/intermediate demand, 20 (6.2%) as early high demand, 20 (6.2%) as late-increasing demand, and 61 (18.8%) as persistent high demand (Figure 2 and Supplementary Table S1). All 61 patients with persistent high demand also met the protocol-specified high 48-h consumption criterion (>38.1 mg). In addition, 9 of 20 patients with early high demand and 8 of 20 patients with late-increasing demand exceeded the 48-h threshold, whereas none of the patients with low/intermediate demand did so.

**Figure 2:**
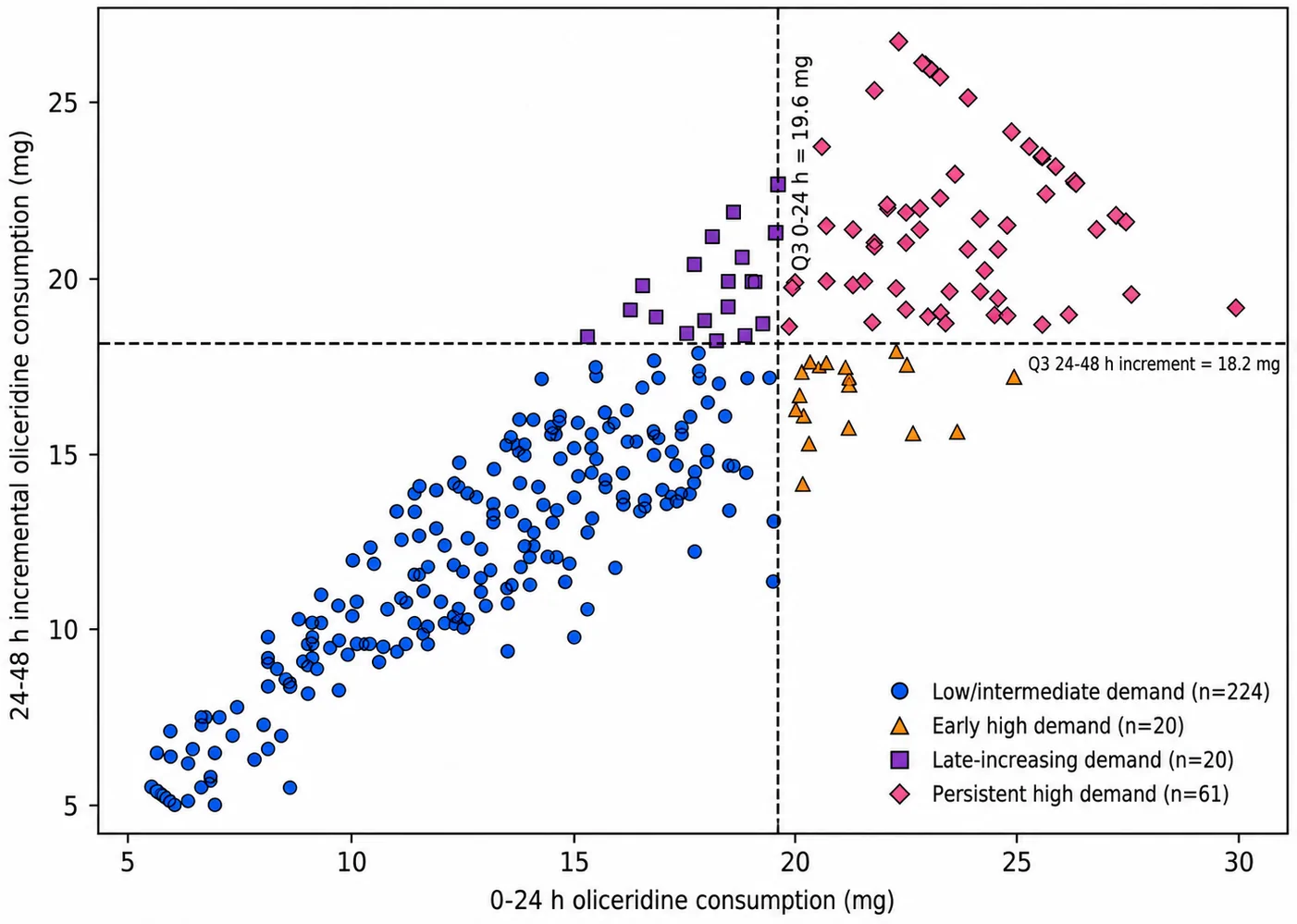
Two-period oliceridine PCIA demand phenotype map. The *x*-axis represents cumulative oliceridine consumption during 0–24 h, and the *y*-axis represents incremental consumption during 24–48 h. The vertical dashed line indicates the third quartile of cumulative 0–24 h consumption (19.6 mg), and the horizontal dashed line indicates the third quartile of incremental 24–48 h consumption (18.2 mg). Patients were classified into four mutually exclusive phenotypes: low/intermediate demand, early high demand, late-increasing demand, and persistent high demand. Points were slightly jittered to reduce overplotting. PCIA, patient-controlled intravenous analgesia; Q3, third quartile.

### 3.2 Baseline and perioperative characteristics

Baseline and perioperative characteristics are summarized in Table 1. Age differed significantly across the four demand phenotypes (global *P* < 0.001). The median age was 42.0 years (IQR, 37.0–51.0) in the persistent high-demand group and 50.0 years (IQR, 40.0–57.0) in the low/intermediate-demand group. No statistically significant global differences were observed in BMI, ASA physical status, hypertension, diabetes mellitus, history of PONV, or disease nature.

**Table 1:** Baseline and perioperative characteristics according to two-period oliceridine demand phenotypes.

Variable	Overall (*N* = 325)	Low/intermediate demand (*n* = 224)	Early high demand (*n* = 20)	Late-increasing demand (*n* = 20)	Persistent high demand (*n* = 61)	*P* value
Age, years	48.0 [39.0, 56.0]	50.0 [40.0, 57.0]	41.0 [31.0, 51.2]	49.0 [40.5, 55.5]	42.0 [37.0, 51.0]	<0.001
BMI, kg/m^^2^	22.9 [20.3, 25.6]	22.7 [20.3, 25.7]	22.1 [19.1, 25.6]	23.7 [21.4, 25.4]	23.4 [20.6, 25.5]	0.738
ASA physical status						0.687
I	82 (25.2)	59 (26.3)	5 (25.0)	2 (10.0)	16 (26.2)	
II	219 (67.4)	150 (67.0)	14 (70.0)	16 (80.0)	39 (63.9)	
III	24 (7.4)	15 (6.7)	1 (5.0)	2 (10.0)	6 (9.8)	
Smoking history	5 (1.5)	5 (2.2)	0 (0.0)	0 (0.0)	0 (0.0)	0.787
Hypertension	72 (22.2)	47 (21.0)	3 (15.0)	4 (20.0)	18 (29.5)	0.462
Diabetes mellitus	37 (11.4)	28 (12.5)	0 (0.0)	2 (10.0)	7 (11.5)	0.440
History of PONV	58 (17.8)	34 (15.2)	5 (25.0)	7 (35.0)	12 (19.7)	0.109
Disease nature						0.710
Benign	161 (49.5)	112 (50.0)	12 (60.0)	9 (45.0)	28 (45.9)	
Malignant	164 (50.5)	112 (50.0)	8 (40.0)	11 (55.0)	33 (54.1)	
Surgical type						0.015
Adnexal surgery	71 (21.8)	54 (24.1)	5 (25.0)	5 (25.0)	7 (11.5)	
Hysterectomy	146 (44.9)	99 (44.2)	6 (30.0)	11 (55.0)	30 (49.2)	
Myomectomy	39 (12.0)	30 (13.4)	4 (20.0)	0 (0.0)	5 (8.2)	
Radical oncologic surgery	48 (14.8)	23 (10.3)	4 (20.0)	4 (20.0)	17 (27.9)	
Other gynecologic surgery	21 (6.5)	18 (8.0)	1 (5.0)	0 (0.0)	2 (3.3)	
Open surgery	72 (22.2)	34 (15.2)	5 (25.0)	8 (40.0)	25 (41.0)	<0.001
Hysterectomy performed	222 (68.3)	144 (64.3)	11 (55.0)	18 (90.0)	49 (80.3)	0.008
Adnexectomy						0.397
None	251 (77.2)	176 (78.6)	14 (70.0)	13 (65.0)	48 (78.7)	
Unilateral	39 (12.0)	24 (10.7)	5 (25.0)	3 (15.0)	7 (11.5)	
Bilateral	35 (10.8)	24 (10.7)	1 (5.0)	4 (20.0)	6 (9.8)	
Lymph node dissection	22 (6.8)	14 (6.2)	1 (5.0)	1 (5.0)	6 (9.8)	0.768
Surgical duration, min	171.0 [136.0, 225.5]	163.0 [125.0, 207.5]	194.0 [164.8, 235.2]	213.5 [154.2, 271.0]	196.0 [168.0, 247.0]	<0.001
Estimated blood loss, mL	30.5 [14.0, 61.8]	28.5 [12.2, 58.8]	29.0 [16.5, 74.0]	27.5 [21.8, 46.5]	40.0 [18.8, 89.0]	0.146
Perioperative blood transfusion	1 (0.3)	0 (0.0)	0 (0.0)	0 (0.0)	1 (1.6)	0.311
Intraoperative dexmedetomidine	132 (40.6)	93 (41.5)	12 (60.0)	9 (45.0)	18 (29.5)	0.091
Intraoperative NSAIDs	248 (76.3)	167 (74.6)	15 (75.0)	16 (80.0)	50 (82.0)	0.680
Regional block	155 (47.7)	117 (52.2)	10 (50.0)	8 (40.0)	20 (32.8)	0.050
Wound infiltration	183 (56.3)	133 (59.4)	13 (65.0)	10 (50.0)	27 (44.3)	0.145

Values are presented as median [interquartile range] or *n* (%). Percentages are column percentages. For binary variables, *n* (%) represents the number and percentage of patients with the characteristic. *P* values compare the four demand-phenotype groups and were calculated using the Kruskal–Wallis test for continuous variables and Pearson's chi-squared test or Fisher's exact test with Monte Carlo simulation using 1,000,000 replicates for categorical variables, as appropriate. Surgical duration was available for 319 patients, and estimated blood loss was available for 318 patients. ASA, American Society of Anesthesiologists; BMI, body mass index; NSAIDs, non-steroidal anti-inflammatory drugs; PONV, postoperative nausea and vomiting.

Surgical characteristics also varied across phenotypes. The distribution of surgical type differed across the four groups (global *P* = 0.015). Open surgery accounted for 41.0% of the persistent high-demand group and 15.2% of the low/intermediate-demand group (global *P* < 0.001). Hysterectomy was performed in 80.3% and 64.3% of patients in these groups, respectively (global *P* = 0.008). Surgical duration differed across phenotypes (global *P* < 0.001), with median durations of 196.0 min (IQR, 168.0–247.0) in the persistent high-demand group and 163.0 min (IQR, 125.0–207.5) in the low/intermediate-demand group.

Regional block was used in 32.8% of patients with persistent high demand and 52.2% of those with low/intermediate demand; the global four-group comparison yielded *P* = 0.050. Intraoperative dexmedetomidine use also varied numerically across groups, but the overall difference was not statistically significant (global *P* = 0.091).

### 3.3 PCIA consumption, demand counts, and postoperative outcomes

PCIA-related measures, postoperative pain scores, APS interventions, and adverse outcomes are summarized in Table 2. As expected from the phenotype definitions, cumulative 48-h oliceridine consumption differed across the four groups (global *P* < 0.001). Median cumulative 48-h consumption was 44.4 mg (IQR, 42.6–49.0) in the persistent high-demand group, compared with 25.5 mg (IQR, 19.4–30.3) in the low/intermediate-demand group, 38.1 mg (IQR, 36.9–38.8) in the early high-demand group, and 37.8 mg (IQR, 36.4–39.1) in the late-increasing-demand group.

**Table 2:** PCIA consumption, demand counts, postoperative pain, APS interventions, and adverse outcomes according to two-period demand phenotypes.

Variable	Overall (*N* = 325)	Low/intermediate demand (*n* = 224)	Early high demand (*n* = 20)	Late-increasing demand (*n* = 20)	Persistent high demand (*n* = 61)	*P* value
0–24 h oliceridine consumption, mg	15.4 [11.5, 19.6]	12.9 [9.6, 15.5]	20.8 [20.2, 22.2]	18.4 [17.7, 18.9]	23.2 [22.0, 24.7]	-
24–48 h incremental oliceridine consumption, mg	14.2 [10.7, 18.2]	12.3 [9.6, 14.5]	17.2 [16.0, 17.6]	19.5 [18.8, 20.5]	21.4 [19.6, 23.0]	-
0–48 h oliceridine consumption, mg	30.1 [22.7, 38.1]	25.5 [19.4, 30.3]	38.1 [36.9, 38.8]	37.8 [36.4, 39.1]	44.4 [42.6, 49.0]	<0.001
48 h effective PCIA demands	9.0 [5.0, 13.0]	7.0 [4.0, 9.0]	13.0 [13.0, 14.0]	13.0 [12.0, 14.0]	17.0 [16.0, 18.0]	<0.001
48 h total PCIA demands	11.0 [7.0, 16.0]	9.0 [6.0, 12.0]	15.0 [13.8, 17.0]	16.0 [15.0, 17.0]	18.0 [18.0, 19.0]	<0.001
POD1 resting NRS	2.0 [1.0, 3.0]	1.0 [0.0, 2.0]	2.0 [1.0, 3.0]	3.0 [2.0, 3.2]	4.0 [3.0, 4.0]	<0.001
POD1 movement NRS	4.0 [3.0, 5.0]	3.0 [2.0, 4.0]	5.0 [5.0, 6.2]	5.0 [4.0, 5.0]	6.0 [5.0, 7.0]	<0.001
POD2 resting NRS	1.0 [0.0, 2.0]	0.0 [0.0, 1.0]	2.0 [1.0, 2.0]	1.0 [1.0, 2.0]	2.0 [2.0, 3.0]	<0.001
POD2 movement NRS	2.0 [2.0, 3.0]	2.0 [1.0, 3.0]	3.0 [2.0, 4.0]	2.5 [2.0, 3.0]	3.0 [2.0, 3.0]	0.035
Rescue analgesia episodes	0.0 [0.0, 0.0]	0.0 [0.0, 0.0]	0.0 [0.0, 1.0]	0.0 [0.0, 0.0]	0.0 [0.0, 0.0]	0.313
Postoperative hospital stay, days	6.0 [4.0, 7.2]	6.0 [4.0, 7.0]	7.0 [5.0, 8.0]	6.0 [4.8, 8.0]	7.0 [4.0, 8.0]	0.109
High 48 h consumption (>Q3)	78 (24.0)	0 (0.0)	9 (45.0)	8 (40.0)	61 (100.0)	<0.001
PCIA parameter adjustment	41 (12.6)	25 (11.2)	2 (10.0)	2 (10.0)	12 (19.7)	0.378
Early PCIA discontinuation	28 (8.6)	22 (9.8)	1 (5.0)	0 (0.0)	5 (8.2)	0.580
PONV within 48 h	99 (30.5)	65 (29.0)	5 (25.0)	4 (20.0)	25 (41.0)	0.193
Rescue analgesia within 48 h	59 (18.2)	42 (18.8)	6 (30.0)	4 (20.0)	7 (11.5)	0.256
Dizziness	43 (13.2)	26 (11.6)	3 (15.0)	2 (10.0)	12 (19.7)	0.393
Somnolence or sedation	45 (13.8)	29 (12.9)	1 (5.0)	2 (10.0)	13 (21.3)	0.253
Pruritus	19 (5.8)	15 (6.7)	1 (5.0)	0 (0.0)	3 (4.9)	0.859
Respiratory depression	1 (0.3)	1 (0.4)	0 (0.0)	0 (0.0)	0 (0.0)	1.000

Values are presented as median [interquartile range] or *n* (%). Percentages are column percentages. *P* values compare the four demand-phenotype groups and were calculated using the Kruskal–Wallis test for continuous variables and Pearson's chi-squared test or Fisher's exact test with Monte Carlo simulation using 1,000,000 replicates for categorical variables, as appropriate. Cumulative 0–24 h consumption and incremental 24–48 h consumption were used to define the phenotype categories and are presented for descriptive interpretation; corresponding inferential *P* values are not reported. No *post hoc* pairwise comparisons were performed. POD1 movement NRS was available for 318 patients, and postoperative length of stay was available for 320 patients. APS, acute pain service; IQR, interquartile range; NRS, numerical rating scale; PCIA, patient-controlled intravenous analgesia; POD, postoperative day; PONV, postoperative nausea and vomiting; Q3, third quartile.

Device-recorded PCIA demand counts also differed across phenotypes (global *P* < 0.001). Patients with persistent high demand had median effective and total PCIA demand counts of 17.0 (IQR, 16.0–18.0) and 18.0 (IQR, 18.0–19.0), respectively, whereas the corresponding values in the low/intermediate-demand group were 7.0 (IQR, 4.0–9.0) and 9.0 (IQR, 6.0–12.0).

Postoperative pain scores were generally higher in the persistent high-demand group. Median POD1 resting NRS was 4.0 (IQR, 3.0–4.0) in the persistent high-demand group and 1.0 (IQR, 0.0–2.0) in the low/intermediate-demand group; the corresponding POD1 movement NRS values were 6.0 (IQR, 5.0–7.0) and 3.0 (IQR, 2.0–4.0). Global differences were also observed for POD2 resting NRS (*P* < 0.001) and POD2 movement NRS (*P* = 0.035).

PONV within 48 h occurred in 41.0% of patients with persistent high demand and 29.0% of those with low/intermediate demand, although the overall four-group difference was not statistically significant (global *P* = 0.193). No statistically significant global differences were observed in rescue analgesia, PCIA parameter adjustment, early PCIA discontinuation, dizziness, somnolence or sedation, pruritus, respiratory depression, or postoperative length of stay.

### 3.4 Protocol-specified primary continuous outcome and exploratory phenotype-specific analyses

The multivariable analysis of the protocol-specified primary continuous outcome is presented in Supplementary Table S2. In the complete-case analysis (*n* = 319), each 10-year increase in age was associated with 2.73 mg lower cumulative 48-h oliceridine consumption (95% CI, −3.47 to −2.00; *P* < 0.001), whereas each 60-min increase in surgical duration was associated with 2.73 mg higher consumption (95% CI, 1.86–3.60; *P* < 0.001). Open surgery and hysterectomy were associated with adjusted mean increases of 6.85 mg (95% CI, 4.65–9.05; *P* < 0.001) and 5.26 mg (95% CI, 3.27–7.25; *P* < 0.001), respectively. Regional block and intraoperative dexmedetomidine were each associated with adjusted mean reductions of 2.58 mg (95% CI, −4.46 to −0.71; *P* = 0.007) and 3.25 mg (95% CI, −5.17 to −1.32; *P* < 0.001), respectively.

The exploratory phenotype-specific and secondary high 48-h consumption logistic regression models are presented in Table 3, and the adjusted associations for the persistent high-demand phenotype are shown in Figure 3. The exploratory phenotype-specific complete-case model included 319 patients, of whom 57 had persistent high demand. Each 10-year increase in age was associated with lower odds of persistent high demand (adjusted OR, 0.56; 95% CI, 0.42–0.74; *P* < 0.001), whereas each 60-min increase in surgical duration was associated with higher odds (adjusted OR, 1.85; 95% CI, 1.35–2.52; *P* < 0.001). Open surgery versus laparoscopy (adjusted OR, 3.67; 95% CI, 1.81–7.45; *P* < 0.001) and hysterectomy performed (adjusted OR, 2.87; 95% CI, 1.32–6.27; *P* = 0.008) were also associated with higher odds of persistent high demand.

**Table 3:** Multivariable logistic regression models for persistent high demand and high 48-h oliceridine consumption.

Variable	Persistent high-demand phenotype OR (95% CI)	*P* value	High 48-h consumption (>38.1 mg) OR (95% CI)	*P* value
Age, per 10-year increase	0.56 (0.42–0.74)	<0.001	0.60 (0.46–0.77)	<0.001
Surgical duration, per 60-min increase	1.85 (1.35–2.52)	<0.001	1.97 (1.47–2.63)	<0.001
Open surgery vs. laparoscopy	3.67 (1.81–7.45)	<0.001	4.08 (2.10–7.94)	<0.001
Hysterectomy performed	2.87 (1.32–6.27)	0.008	2.79 (1.38–5.62)	0.004
Regional block	0.41 (0.21–0.80)	0.009	0.42 (0.23–0.78)	0.006
Intraoperative dexmedetomidine	0.48 (0.24–0.96)	0.037	0.55 (0.30–1.02)	0.056
Model n/events	319/57		319/74	
Apparent C-statistic	0.803		0.796	
Apparent Brier score	0.118		0.140	

Adjusted odds ratios (ORs) with 95% confidence intervals (CIs) are presented. Both complete-case models included age, surgical duration, open surgery versus laparoscopy, hysterectomy performed, regional block, and intraoperative dexmedetomidine. Age and surgical duration were scaled per 10-year and 60-min increases, respectively. Reference categories were laparoscopy, no hysterectomy, no regional block, and no intraoperative dexmedetomidine. Persistent high demand was defined as concurrent 0–24 h cumulative consumption >19.6 mg and 24–48 h incremental consumption >18.2 mg. High 48-h consumption was defined as cumulative 48-h oliceridine consumption >38.1 mg. The C-statistics and Brier scores are apparent performance measures derived from the same cohort used for model estimation, without internal resampling or external validation. Accordingly, these measures may be optimistic and should not be interpreted as validated predictive performance. CI, confidence interval; OR, odds ratio.

**Figure 3:**
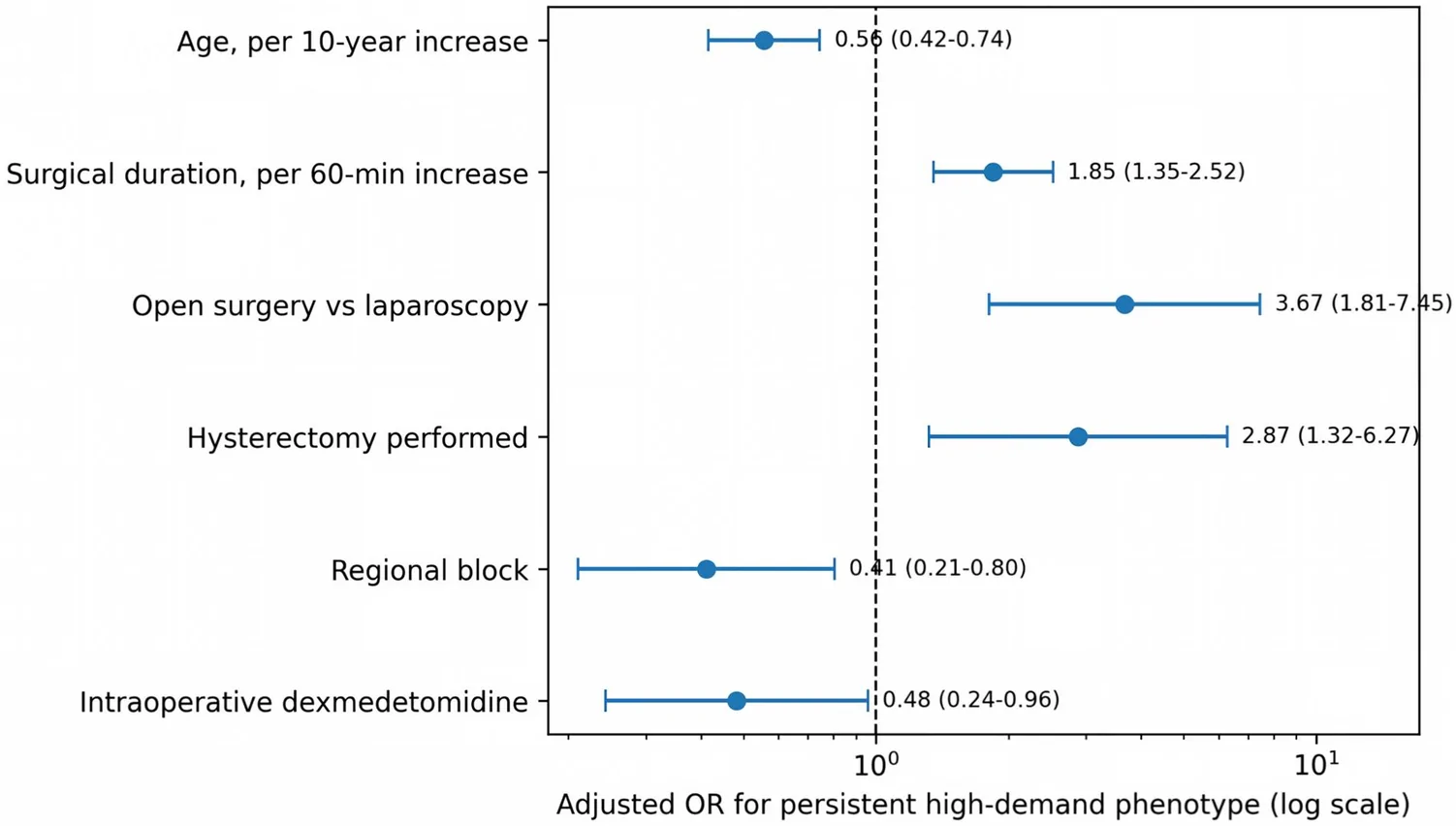
Adjusted perioperative correlates of the persistent high-demand phenotype. The forest plot presents adjusted odds ratios and 95% confidence intervals from the exploratory phenotype-specific complete-case multivariable logistic regression model. The model included 319 patients, of whom 57 had persistent high demand, and simultaneously included age, surgical duration, open surgery versus laparoscopy, hysterectomy performed, regional block, and intraoperative dexmedetomidine. Age and surgical duration were modeled per 10-year and 60-min increases, respectively. Reference categories were laparoscopy, no hysterectomy, no regional block, and no intraoperative dexmedetomidine. The vertical dashed line denotes an odds ratio of 1. CI, confidence interval; OR, odds ratio.

Regional block (adjusted OR, 0.41; 95% CI, 0.21–0.80; *P* = 0.009) and intraoperative dexmedetomidine (adjusted OR, 0.48; 95% CI, 0.24–0.96; *P* = 0.037) were associated with lower odds of persistent high demand. No problematic multicollinearity was observed among the model covariates (maximum variance inflation factor, 1.02). The apparent C-statistic of the exploratory phenotype-specific model was 0.803, and the Brier score was 0.118.

The secondary model using the protocol-specified high 48-h oliceridine consumption outcome (>38.1 mg) included 319 patients and 74 events. The directions and approximate magnitudes of the associations for age, surgical duration, open surgery, hysterectomy, and regional block were consistent with those observed in the exploratory phenotype-specific model. The association for intraoperative dexmedetomidine was directionally similar but did not reach statistical significance (adjusted OR, 0.55; 95% CI, 0.30–1.02; *P* = 0.056). The apparent C-statistic and Brier score of the secondary model were 0.796 and 0.140, respectively.

### 3.5 Associations of multimodal analgesia combinations with oliceridine demand

Associations between regional block/dexmedetomidine combinations and oliceridine demand are summarized in Table 4 and Figure 4. Persistent high demand occurred in 28 of 104 patients (26.9%) who received neither intervention, 13 of 66 (19.7%) who received dexmedetomidine only, 15 of 89 (16.9%) who received regional block only, and 5 of 66 (7.6%) who received both regional block and dexmedetomidine. The protocol-specified high 48-h consumption outcome showed a similar numerical pattern, with corresponding rates of 32.7%, 25.8%, 21.3%, and 12.1%.

**Table 4:** Associations of regional block/dexmedetomidine combinations with oliceridine demand.

Analgesia group	n	Persistent high demand n (%)	High 48-h consumption (>38.1 mg) n (%)	48 h consumption median [IQR]	Adjusted OR for persistent high demand (95% CI)	*P* value	Adjusted mean difference in cumulative 48-h consumption, mg (95% CI)	*P* value
Neither regional block nor dexmedetomidine	104	28 (26.9)	34 (32.7)	32.4 [24.0, 41.9]	Reference		Reference	
Dexmedetomidine only	66	13 (19.7)	17 (25.8)	30.2 [21.5, 38.2]	0.61 (0.26–1.44)	0.257	−2.8 (−5.6 to −0.1)	0.045
Regional block only	89	15 (16.9)	19 (21.3)	30.2 [25.2, 35.2]	0.50 (0.22–1.12)	0.092	−2.2 (−4.7 to 0.2)	0.072
Regional block + dexmedetomidine	66	5 (7.6)	8 (12.1)	26.7 [19.8, 33.3]	0.17 (0.05–0.50)	0.002	−5.9 (−8.7 to −3.1)	<0.001

Values in the descriptive columns are presented as *n* (%) or median [interquartile range]. Raw descriptive data included all 325 patients, whereas the adjusted models were based on 319 complete cases. The reference group comprised patients who received neither regional block nor intraoperative dexmedetomidine. Adjusted ORs for persistent high demand were estimated using multivariable logistic regression, and adjusted mean differences in cumulative 48-h oliceridine consumption were estimated using linear regression with heteroscedasticity-consistent type 3 (HC3) robust standard errors. Both adjusted models included age, surgical duration, open surgery, and hysterectomy. *P* values correspond to the adjusted models. Negative adjusted mean differences indicate lower cumulative 48-h consumption than in the reference group. CI, confidence interval; HC3, heteroscedasticity-consistent type 3; IQR, interquartile range; OR, odds ratio. As a multiplicity sensitivity analysis, Holm-adjusted *P* values were calculated across the three contrasts with the reference group separately for each adjusted outcome. For persistent high demand, Holm-adjusted *P* values were 0.257, 0.185, and 0.005 for dexmedetomidine only, regional block only, and regional block plus dexmedetomidine, respectively. For cumulative 48-h consumption, the corresponding Holm-adjusted *P* values were 0.092, 0.092, and <0.001.

**Figure 4:**
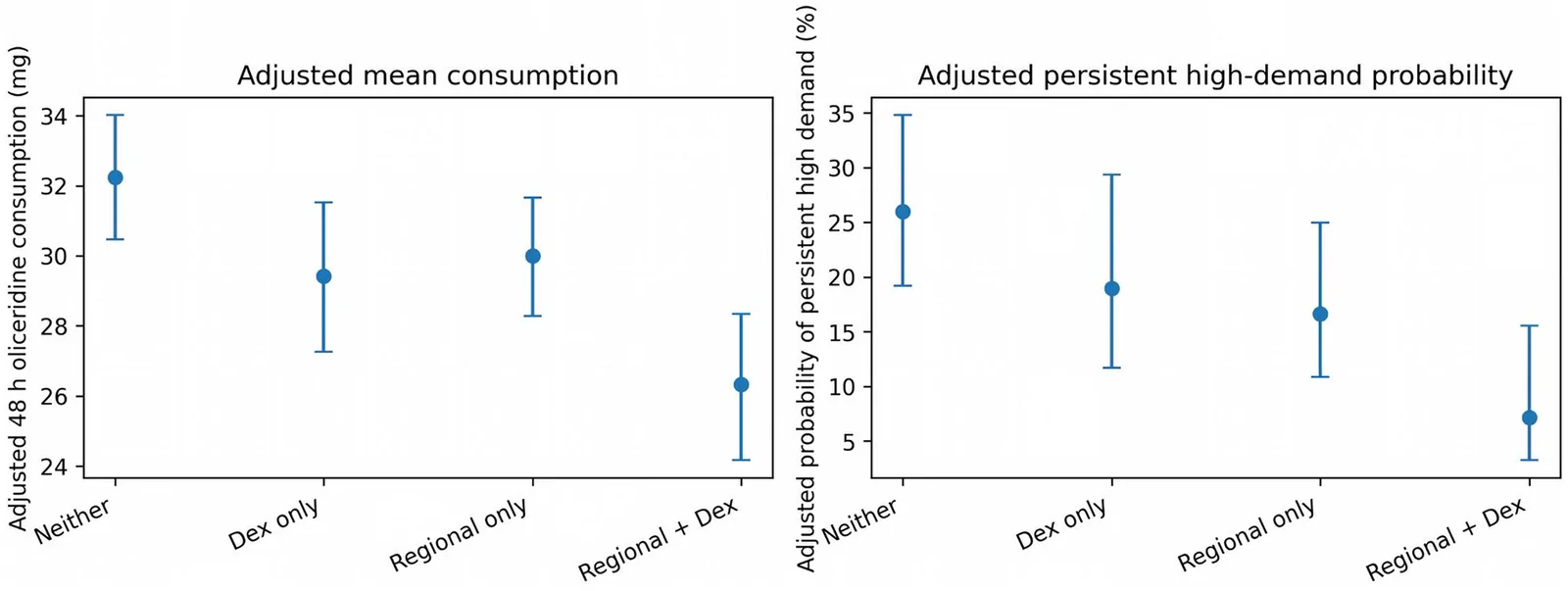
Multimodal analgesia combinations and adjusted oliceridine demand. Patients were classified into four mutually exclusive groups according to receipt of regional block and intraoperative dexmedetomidine: neither intervention, dexmedetomidine only, regional block only, or both interventions. **(A)** Model-based adjusted mean cumulative 48-h oliceridine consumption. **(B)** Model-based adjusted probability of the persistent high-demand phenotype. Both models were based on 319 complete cases and adjusted for age, surgical duration, open surgery, and hysterectomy. Adjusted means and probabilities were standardized over the observed covariate distribution. Points represent adjusted estimates, and error bars represent 95% confidence intervals. CI, confidence interval.

After adjustment for age, surgical duration, open surgery, and hysterectomy, receipt of both regional block and dexmedetomidine was associated with lower odds of persistent high demand compared with receipt of neither intervention (adjusted OR, 0.17; 95% CI, 0.05–0.50; *P* = 0.002). Dexmedetomidine only (adjusted OR, 0.61; 95% CI, 0.26–1.44; *P* = 0.257) and regional block only (adjusted OR, 0.50; 95% CI, 0.22–1.12; *P* = 0.092) showed estimates in the same direction, although their confidence intervals included the null value.

In the adjusted continuous-outcome model, the combined regional block plus dexmedetomidine group had lower 48-h oliceridine consumption than the reference group (adjusted mean difference, −5.9 mg; 95% CI, −8.7 to −3.1; *P* < 0.001). The adjusted mean difference for dexmedetomidine only was −2.8 mg (95% CI, −5.6 to −0.1; *P* = 0.045), whereas the estimate for regional block only was −2.2 mg (95% CI, −4.7 to 0.2; *P* = 0.072). After Holm adjustment across the three contrasts with the reference group within each outcome, the association for combined regional block plus dexmedetomidine remained statistically significant for both persistent high demand (Holm-adjusted *P* = 0.005) and cumulative 48-h oliceridine consumption (Holm-adjusted *P* < 0.001). The dexmedetomidine-only and regional-block-only contrasts were not statistically significant after adjustment (Holm-adjusted *P* = 0.257 and 0.185, respectively, for persistent high demand; and *P* = 0.092 and 0.092, respectively, for cumulative 48-h consumption).

### 3.6 Sensitivity analyses

Across the sensitivity analyses, the directions and approximate magnitudes of the associations in the exploratory phenotype-specific model remained broadly consistent (Supplementary Table S3). Similar estimates were observed after excluding patients with any early PCIA discontinuation, non-depletion-related discontinuation, unexplained low-consumption records, or PCIA parameter adjustment, and in the strict analysis set. However, confidence intervals widened in some analyses, particularly for intraoperative dexmedetomidine. After exclusion of patients with cumulative 48-h consumption of 49 mg, the associations for age, surgical duration, and open surgery remained evident, whereas the estimates for hysterectomy, regional block, and dexmedetomidine were attenuated and their 95% CIs included the null value.

The distributions of cumulative 0–24 h oliceridine consumption, incremental 24–48 h consumption, and cumulative 0–48 h consumption are shown in Supplementary Figure S1.

## 4 Discussion

### 4.1 Principal findings

In the protocol-specified primary analysis, younger age, longer surgical duration, open surgery, and hysterectomy were associated with greater cumulative 48-h oliceridine consumption, whereas regional block and intraoperative dexmedetomidine were each associated with lower consumption. Exploratory *post hoc* classification identified four clinically interpretable two-period phenotypes; approximately one in five patients had persistent high demand. Phenotype-specific associations were broadly concordant with the continuous-outcome findings. Patients receiving both regional block and dexmedetomidine had the lowest adjusted probability of persistent high demand and the lowest adjusted 48-h consumption, although these treatment-selection associations should not be interpreted causally.

The two-period phenotype framework added temporal resolution to a single cumulative-dose endpoint. Although all patients with persistent high demand exceeded the 48-h Q3 threshold, patients with early high demand and those with late-increasing demand had similar cumulative 48-h consumption but differed in when that consumption occurred. This distinction may be relevant to APS planning because early-predominant, late-increasing, and sustained demand patterns may warrant reassessment and modification of multimodal analgesia at different postoperative time points.

### 4.2 Interpretation of the two-period demand phenotypes

Postoperative opioid consumption is commonly summarized as a cumulative dose over a fixed interval, but similar totals may arise from different temporal patterns (14–16). By separating 0–24 h cumulative consumption from the 24–48 h incremental dose, the present rule-based classification distinguished patients with predominantly early demand from those whose requirements increased later. The early high-demand and late-increasing groups had nearly identical median 48-h consumption—38.1 and 37.8 mg, respectively—despite differing in how consumption was distributed across the two periods. Thus, the two-period framework provided descriptive information that was not captured by a single 48-h threshold.

The persistent high-demand phenotype represented sustained high consumption across both periods. Greater PCIA demand counts were expected because successful bolus delivery contributed directly to cumulative consumption; the accompanying higher postoperative NRS scores provided additional clinical context but did not constitute independent validation of the phenotype. These measures were recorded within the same 48-h outcome window and may reflect both underlying nociceptive burden and responses to analgesic treatment. Accordingly, the four groups should be regarded as exploratory descriptive phenotypes rather than biologically distinct subtypes or formal longitudinal trajectories. Because the Q3 thresholds were derived from the same cohort in which the phenotype associations were evaluated, both the thresholds and the resulting phenotype distribution may be sample-specific. They should therefore not be interpreted as validated clinical cutoffs and require replication in independent cohorts. Postoperative pain scores and device-demand variables were therefore treated as postoperative outcomes rather than baseline predictors.

### 4.3 Patient characteristics and surgical burden

Age showed a consistent inverse association with oliceridine demand across the persistent high-demand, protocol-specified high-consumption, and continuous-consumption analyses. This finding is broadly consistent with previous evidence that younger adults frequently report greater acute postoperative pain and consume more opioid analgesics than older adults (1–3). Potential explanations include age-related differences in nociceptive processing, opioid pharmacodynamics, drug disposition, expectations, and pain-reporting behavior. However, these mechanisms were not directly measured in the present study, and age should therefore be interpreted as a clinical risk marker rather than a mechanistic determinant of oliceridine demand.

Longer surgical duration, open surgery, and hysterectomy were each associated with persistent high demand after adjustment and showed concordant associations in the continuous-consumption analysis. These variables may represent complementary dimensions of surgical burden, including procedure duration, operative approach, and extent of tissue resection. Compared with minimally invasive procedures, open surgery generally involves greater tissue disruption, whereas hysterectomy and prolonged operations may entail broader dissection and more sustained nociceptive input (1–3). The consistency of these findings across binary and continuous outcomes suggests that the observed associations were unlikely to be explained solely by the Q3-based phenotype definition.

Nevertheless, surgical duration and procedure selection are also related to disease severity, technical complexity, surgeon preference, and other perioperative factors that were not fully captured in the dataset. The observed associations should therefore be regarded as signals for perioperative risk stratification rather than causal effects of specific procedures.

### 4.4 Modifiable perioperative analgesic correlates

Regional block and intraoperative dexmedetomidine showed consistent inverse associations across the protocol-specified continuous-outcome analysis and the exploratory phenotype-specific model. In the four-group analysis, patients receiving both interventions had the lowest observed proportion of persistent high demand. Compared with neither intervention, combined use was associated with lower odds of persistent high demand (adjusted OR, 0.17; 95% CI, 0.05–0.50) and 5.9 mg lower adjusted 48-h consumption. Both contrasts remained significant after Holm adjustment, whereas the single-intervention contrasts did not. These findings are clinically plausible: regional techniques are established components of multimodal pain management, although their opioid-sparing effects vary by surgical approach and block technique (18–22), and evidence supports an opioid-sparing role for perioperative dexmedetomidine that may depend on dose, timing, and administration regimen (23–25).

Nevertheless, these findings should not be interpreted as evidence of synergy or a causal treatment effect. Regional block and dexmedetomidine were selected by the attending anesthesiologist rather than randomly assigned, and treatment decisions may have been influenced by anticipated pain intensity, operative complexity, patient characteristics, or clinician preference. The direction of confounding by indication is uncertain. Clinicians may have preferentially selected multimodal interventions for patients expected to experience greater postoperative pain, which could attenuate an apparent opioid-sparing association. Conversely, regional techniques or combined multimodal strategies may have been more readily used in patients considered clinically suitable or hemodynamically stable, which could bias the observed association in the opposite direction. A treatment-selection effect analogous to healthy-user bias therefore cannot be excluded.

Although routine institutional practice predominantly involved ultrasound-guided transversus abdominis plane blocks using ropivacaine without routinely added block adjuvants, patient-level details regarding the exact block type, local-anesthetic concentration and volume, and adjuvant use were not consistently available. Dexmedetomidine doses and infusion durations were also unavailable, and the four-group analysis did not constitute a formal statistical test of interaction. The estimates for either intervention alone were directionally favorable but less precise, with confidence intervals for the persistent high-demand outcome including the null value. Residual confounding and treatment-selection bias may therefore have influenced the observed associations. Accordingly, the combination analysis should be regarded as exploratory and hypothesis-generating rather than as evidence of additive or synergistic benefit. Prospective studies using standardized intervention protocols, detailed dose and technique documentation, and prespecified interaction analyses are needed to evaluate these associations further.

### 4.5 Clinical implications and future directions

The identified preoperative and intraoperative correlates may help clinicians recognize patients who require closer postoperative analgesic surveillance. Younger patients and those undergoing longer procedures, open surgery, or hysterectomy may be more likely to have sustained oliceridine demand. However, these associative models were developed and evaluated in the same cohort and should not be used as clinical prediction tools or treatment algorithms. A recent network meta-analysis found that organized APS models provided better postoperative pain relief than traditional ward-based care (26).

The two-period phenotype framework may have a complementary role. Within a structured APS pathway, unexpectedly high 0–24 h consumption may signal the need for early reassessment of pain control, PCIA use, and multimodal coverage. Late-increasing and persistent high-demand phenotypes, however, can be fully characterized only after the second postoperative period and should remain descriptive rather than real-time decision rules. The findings do not support routine escalation of initial PCIA settings; instead, unexpectedly high or sustained consumption may prompt structured reassessment and individualized optimization of multimodal analgesia. This use is consistent with postoperative pain guidelines, organized APS models, and Enhanced Recovery After Surgery principles in gynecologic surgery (4–6, 18, 26–28).

Future multicenter prospective studies should evaluate whether the two-period thresholds and phenotype distribution are reproducible in other surgical populations and clinical settings. Such studies should prospectively capture complete pump-derived cumulative remifentanil dose and event-level infusion data, together with event-level PCIA data and repeated pain and adverse-event assessments; standardize regional block techniques and dexmedetomidine dosing; and prespecify analyses of possible additive or interaction effects. If these phenotypes are subsequently incorporated into a clinical risk model, internal and external validation, calibration assessment, and evaluation of clinical utility will be required. Ultimately, randomized or protocol-based implementation studies are needed to determine whether phenotype-informed APS management improves pain control, reduces opioid-related adverse events, and enhances postoperative recovery.

### 4.6 Strengths and limitations

Several features strengthen this study. Oliceridine consumption was obtained from PCIA device records rather than inferred from prescriptions, and separating 0–24 h from 24 to 48 h consumption added temporal information beyond the protocol-specified 48-h endpoint. Both the primary continuous-outcome model and the exploratory phenotype-specific model used a limited set of clinically selected preoperative and intraoperative covariates, avoiding automated stepwise selection and adjustment for postoperative mediators or consequences. Robustness was assessed using the protocol-specified high-consumption outcome and sensitivity analyses addressing early discontinuation, unexplained low consumption, reservoir-ceiling effects, and PCIA parameter adjustment.

Several limitations should be acknowledged. First, the single-center retrospective design limits external generalizability and precludes causal inference. Retrospective registration after data collection also limits independent verification of prospective specification of the study outcomes and exploratory analyses. Second, the two-period phenotypes were based on cohort-specific third-quartile thresholds derived and evaluated in the same dataset and on only two cumulative assessment times. The classification is therefore data-driven and potentially sample-specific; the thresholds and phenotype distribution require replication in independent cohorts before clinical application. The early high-demand and late-increasing groups each contained only 20 patients, limiting the precision of comparisons involving these phenotypes.

Third, several potentially relevant covariates were unavailable, including cumulative intraoperative opioid exposure, preoperative pain, anxiety, pain catastrophizing, sleep quality, and socioeconomic characteristics. Although the induction dose of sufentanil and institutional remifentanil target range were protocolized, complete pump-derived remifentanil exposure could not be consistently retrieved or validly reconstructed. Residual variation in remifentanil exposure may therefore have confounded associations with postoperative oliceridine consumption. Fourth, detailed patient-level information on regional block technique, local-anesthetic regimen, adjuvant use, and dexmedetomidine dose and duration was unavailable. Because these interventions were clinician-selected rather than randomized, residual confounding, confounding by indication, and treatment-selection or healthy-user-type bias cannot be excluded. The four-group analysis was not a formal interaction test and cannot establish additive or synergistic effects.

Fifth, the 50-mg PCIA reservoir imposed a potential upper ceiling on observed consumption, whereas early discontinuation and PCIA parameter adjustment may have altered the recorded dose independently of underlying analgesic requirements. Although corresponding sensitivity analyses were conducted, they cannot fully eliminate measurement-related bias. Sixth, the study did not include a comparator group receiving a conventional opioid and therefore cannot address the comparative analgesic effectiveness or safety of oliceridine. Finally, the exploratory phenotype-specific complete-case model included 57 persistent high-demand events and six prespecified covariates, corresponding to approximately 9.5 events per covariate. Although model complexity was deliberately restricted and no automated variable-selection procedure was used, some degree of overfitting cannot be excluded. The reported C-statistic and Brier score were apparent in-sample performance measures derived from the same cohort used for model estimation and may therefore be optimistic. These measures should not be interpreted as validated predictive performance, and future studies should incorporate internal resampling and external validation. In addition, the physicochemical compatibility and stability of the oliceridine–dolasetron admixture were not independently evaluated; therefore, potential admixture-related effects cannot be fully excluded.

## 5 Conclusion

In this cohort, younger age, longer surgical duration, open surgery, and hysterectomy were associated with greater cumulative 48-h oliceridine consumption, whereas regional block and intraoperative dexmedetomidine were each associated with lower consumption. In exploratory *post hoc* analyses, the combination of regional block and intraoperative dexmedetomidine showed the largest adjusted reduction in 48-h oliceridine consumption and the lowest odds of persistent high demand compared with neither intervention; these combined-intervention contrasts remained statistically significant after Holm adjustment, whereas the single-intervention contrasts did not. This observational finding is hypothesis-generating and requires prospective confirmation, including formal evaluation of whether the combined effect is additive or synergistic. Exploratory two-period phenotypes added temporal context and identified a persistent high-demand subgroup. These findings require prospective multicenter validation before use in clinical decision-making.

## Data Availability

The de-identified data supporting the findings of this study are not publicly available because they contain clinical information subject to institutional and ethical restrictions. A de-identified analytic dataset and statistical code may be made available by the corresponding author upon reasonable request and with approval from the relevant institutional authorities.
