# Supplementary material for: Two-period demand phenotypes of oliceridine-based patient-controlled intravenous analgesia after gynecologic surgery: perioperative correlates of persistent high demand
Source: Front Med (Lausanne). 2026 Oct 14;13:1970542. doi: 10.3389/fmed.2026.1970542 (PMC13713527; doi:10.3389/fmed.2026.1970542)
Supplement: Supplementary file 1 [file Datasheet1.pdf]

STROBE Statement—Checklist of items that should be included in reports of cohort studies

Manuscript: Two-period demand phenotypes of oliceridine-based patient-controlled intravenous analgesia after gynecologic surgery: perioperative correlates of persistent high demand

Article type: Original Research — retrospective cohort study

Section/topic	Item	Recommendation	Reported on page/section
**Title and abstract**	**1(a)**	Indicate the study's design with a commonly used term in the title or the abstract.	Abstract, p. 2 (single-center retrospective cohort study)
	**1(b)**	Provide in the abstract an informative and balanced summary of what was done and what was found.	Abstract, p. 2
**Introduction — Background/rationale**	**2**	Explain the scientific background and rationale for the investigation being reported.	Introduction, pp. 2–3
**Introduction — Objectives**	**3**	State specific objectives, including any prespecified hypotheses.	Introduction, p. 3
**Methods — Study design**	**4**	Present key elements of study design early in the paper.	Section 2.1, p. 3
**Methods — Setting**	**5**	Describe the setting, locations, and relevant dates, including periods of recruitment, exposure, follow-up, and data collection.	Sections 2.1–2.3, pp. 3–4
**Methods — Participants**	**6(a)**	For a cohort study, give the eligibility criteria and the sources and methods of selection of participants. Describe methods of follow-up.	Section 2.2, p. 3; 48-h observation and APS follow-up, pp. 4–5
	**6(b)**	For matched studies, give matching criteria and number of exposed and unexposed participants.	Not applicable — the cohort was not matched
**Methods — Variables**	**7**	Clearly define all outcomes, exposures, predictors, potential confounders, and effect modifiers. Give diagnostic criteria, if applicable.	Sections 2.4–2.6, pp. 4–6
**Methods — Data sources/measurement**	**8**	For each variable of interest, give sources of data and details of methods of assessment (measurement). Describe comparability of assessment methods if there is more than one group.	Sections 2.1, 2.3 and 2.4, pp. 3–5
**Methods — Bias**	**9**	Describe any efforts to address potential sources of bias.	Sections 2.2 and 2.7, pp. 3 and 6–7; Section 4.6, pp. 11–12
**Methods — Study size**	**10**	Explain how the study size was arrived at.	Section 2.7, p. 6 (all eligible patients during the prespecified period; no formal sample-size calculation)
**Methods — Quantitative variables**	**11**	Explain how quantitative variables were handled in the analyses. If applicable, describe which groupings were chosen and why.	Sections 2.5–2.7, pp. 5–7
**Methods — Statistical methods**	**12(a)**	Describe all statistical methods, including those used to control for confounding.	Section 2.7, pp. 6–7
	**12(b)**	Describe any methods used to examine subgroups and interactions.	Section 2.7, p. 6 (four-group regional block/dexmedetomidine analysis; no formal interaction claim)
	**12(c)**	Explain how missing data were addressed.	Section 2.7, p. 6 (complete-case analyses; no imputation because covariate missingness was <5%)
	**12(d)**	If applicable, explain how loss to follow-up was addressed.	Sections 2.2 and 2.7, pp. 3 and 6–7 (early PCIA discontinuation and corresponding sensitivity analyses)
	**12(e)**	Describe any sensitivity analyses.	Section 2.7, pp. 6–7; Section 3.6, p. 9; Supplementary Table S3
**Results — Participants**	**13(a)**	Report numbers of individuals at each stage of the study—e.g., potentially eligible, examined for eligibility, confirmed eligible, included, and analyzed.	Section 3.1, p. 7; Figure 1

Section/topic	Item	Recommendation	Reported on page/section
	**13(b)**	Give reasons for non-participation at each stage.	Section 3.1, p. 7; Figure 1
	**13(c)**	Consider use of a flow diagram.	Figure 1 (referenced in Section 3.1, p. 7)
**Results — Descriptive data**	**14(a)**	Give characteristics of study participants and information on exposures and potential confounders.	Section 3.2, p. 7; Table 1, p. 15
	**14(b)**	Indicate the number of participants with missing data for each variable of interest.	Table 1 footnote, p. 15; Table 2 footnote, p. 16
	**14(c)**	Summarize follow-up time (e.g., average and total amount).	Fixed 48-h postoperative observation window described in Sections 2.2–2.5, pp. 3–5
**Results — Outcome data**	**15**	Report numbers of outcome events or summary measures over time.	Sections 3.1 and 3.3, pp. 7–8; Table 2, p. 16
**Results — Main results**	**16(a)**	Give unadjusted estimates and, if applicable, confounder-adjusted estimates and their precision (e.g., 95% confidence interval). Make clear which confounders were adjusted for and why they were included.	Sections 3.4–3.5, pp. 8–9; Tables 3–4, p. 17; Supplementary Table S2
	**16(b)**	Report category boundaries when continuous variables were categorized.	Sections 2.5–2.6, p. 5; Supplementary Table S1
	**16(c)**	If relevant, consider translating estimates of relative risk into absolute risk for a meaningful time period.	Not applicable — the principal adjusted results are mean differences and odds ratios; adjusted probabilities are shown for the exploratory combination analysis
**Results — Other analyses**	**17**	Report other analyses done—e.g., subgroup, interaction, and sensitivity analyses.	Sections 3.5–3.6, p. 9; Supplementary Tables S2–S3
**Discussion — Key results**	**18**	Summarize key results with reference to study objectives.	Section 4.1, p. 9
**Discussion — Limitations**	**19**	Discuss limitations of the study, taking into account sources of potential bias or imprecision. Discuss both direction and magnitude of any potential bias.	Section 4.6, pp. 11–12
**Discussion — Interpretation**	**20**	Give a cautious overall interpretation of results considering objectives, limitations, multiplicity of analyses, results from similar studies, and other relevant evidence.	Sections 4.1–4.6, pp. 9–12
**Discussion — Generalisability**	**21**	Discuss the generalisability (external validity) of the study results.	Sections 4.5–4.6, pp. 10–12
**Other information — Funding**	**22**	Give the source of funding and the role of the funders for the present study and, if applicable, for the original study on which the present article is based.	Funding statement, p. 12 (no external funding)
