# Supplementary material for: Two-period demand phenotypes of oliceridine-based patient-controlled intravenous analgesia after gynecologic surgery: perioperative correlates of persistent high demand
Source: Front Med (Lausanne). 2026 Oct 14;13:1970542. doi: 10.3389/fmed.2026.1970542 (PMC13713527; doi:10.3389/fmed.2026.1970542)
Supplement: Supplementary file 2 [file Table1.docx]

## **Table S1. Definitions and distribution of the two-period oliceridine demand phenotypes.**

| **Phenotype** | **Definition** | **n (%)** | **High 48-h consumption (>38.1 mg) n (%)** | **Cumulative 48-h consumption, median [IQR]** |
| --- | --- | --- | --- | --- |
| Low/intermediate demand | 0-24 h consumption ≤19.6 mg and 24-48 h increment ≤ 18.2 mg | 224 (68.9) | 0 (0.0) | 25.5 [19.4, 30.3] |
| Early high demand | 0-24 h consumption > 19.6 mg and 24-48 h increment≤ 18.2 mg | 20 (6.2) | 9 (45.0) | 38.1 [36.9, 38.8] |
| Late-increasing demand | 0-24 h consumption ≤19.6 mg and 24-48 h increment > 18.2 mg | 20 (6.2) | 8 (40.0) | 37.8 [36.4, 39.1] |
| Persistent high demand | 0-24 h consumption > 19.6 mg and 24-48 h increment > 18.2 mg | 61 (18.8) | 61 (100.0) | 44.4 [42.6, 49.0] |

Values are presented as n (%) or median [interquartile range]. High early demand was defined as cumulative 0–24 h oliceridine consumption >19.6 mg, and high late-period demand was defined as incremental 24–48 h consumption >18.2 mg. Values equal to the respective third-quartile thresholds were classified as not high. Persistent high demand was defined as meeting both the high early-demand and high late-period-demand criteria. Protocol-specified high 48-h consumption was defined as cumulative 48-h oliceridine consumption >38.1 mg. All thresholds were derived from the overall cohort using the type 7 sample-quantile estimator in R. IQR, interquartile range; Q3, third quartile.
