# Supplementary material for: Two-period demand phenotypes of oliceridine-based patient-controlled intravenous analgesia after gynecologic surgery: perioperative correlates of persistent high demand
Source: Front Med (Lausanne). 2026 Oct 14;13:1970542. doi: 10.3389/fmed.2026.1970542 (PMC13713527; doi:10.3389/fmed.2026.1970542)
Supplement: Supplementary file 3 [file Table2.docx]

## **Table S2. Multivariable continuous-outcome model for cumulative 48-h oliceridine consumption.**

| **Variable** | **Adjusted mean difference, mg** | **95% CI** | ***P* value** |
| --- | --- | --- | --- |
| Age, per 10-year increase | -2.73 | -3.47 to -2.00 | <0.001 |
| Surgical duration, per 60-min increase | 2.73 | 1.86 to 3.60 | <0.001 |
| Open surgery vs laparoscopy | 6.85 | 4.65 to 9.05 | <0.001 |
| Hysterectomy performed | 5.26 | 3.27 to 7.25 | <0.001 |
| Regional block | -2.58 | -4.46 to -0.71 | 0.007 |
| Intraoperative dexmedetomidine | -3.25 | -5.17 to -1.32 | <0.001 |
| Model n | 319 |  |  |
| R^2^/ adjusted R^2^ | 0.338 / 0.326 |  |  |

Adjusted mean differences in cumulative 48-h oliceridine consumption are presented with 95% confidence intervals. The complete-case model included 319 patients and adjusted for age, surgical duration, open surgery versus laparoscopy, hysterectomy performed, regional block, and intraoperative dexmedetomidine. Age and surgical duration were scaled per 10-year and 60-min increases, respectively. Reference categories were laparoscopy, no hysterectomy, no regional block, and no intraoperative dexmedetomidine. Estimates were obtained using multivariable linear regression with heteroscedasticity-consistent type 3 (HC3) robust standard errors. Positive values indicate higher cumulative 48-h consumption, whereas negative values indicate lower consumption. The R² and adjusted R² are apparent in-sample measures. CI, confidence interval; HC3, heteroscedasticity-consistent type 3.
