# Supplementary material for: Two-period demand phenotypes of oliceridine-based patient-controlled intravenous analgesia after gynecologic surgery: perioperative correlates of persistent high demand
Source: Front Med (Lausanne). 2026 Oct 14;13:1970542. doi: 10.3389/fmed.2026.1970542 (PMC13713527; doi:10.3389/fmed.2026.1970542)
Supplement: Supplementary file 4 [file Table3.docx]

## **Table S3. Sensitivity analyses of the multivariable model for persistent high demand.**

| **Analysis set** | **n** | **Events** | **Age per 10 yr** | **Surgical duration per 60 min** | **Open surgery** | **Hysterectomy** | **Regional block** | **Dexmedetomidine** |
| --- | --- | --- | --- | --- | --- | --- | --- | --- |
| Main complete-case analysis | 319 | 57 | 0.56 (0.42-0.74) | 1.85 (1.35-2.52) | 3.67 (1.81-7.45) | 2.87 (1.32-6.27) | 0.41 (0.21-0.80) | 0.48 (0.24-0.96) |
| Excluding any early PCIA discontinuation | 292 | 53 | 0.55 (0.40-0.74) | 1.84 (1.33-2.54) | 3.73 (1.79-7.79) | 2.74 (1.21-6.19) | 0.50 (0.25-0.99) | 0.50 (0.25-1.01) |
| Excluding non-depletion-related early discontinuation | 294 | 55 | 0.54 (0.40-0.73) | 1.84 (1.34-2.54) | 3.86 (1.86-8.00) | 2.89 (1.28-6.54) | 0.47 (0.24-0.92) | 0.48 (0.24-0.96) |
| Excluding unexplained low-consumption records | 310 | 57 | 0.56 (0.42-0.75) | 1.83 (1.34-2.50) | 3.59 (1.77-7.29) | 2.84 (1.30-6.20) | 0.41 (0.21-0.81) | 0.49 (0.25-0.97) |
| Excluding 49-mg ceiling-dose records | 303 | 41 | 0.58 (0.42-0.79) | 1.62 (1.15-2.27) | 2.76 (1.26-6.08) | 1.89 (0.85-4.17) | 0.49 (0.24-1.01) | 0.55 (0.26-1.16) |
| Excluding PCIA parameter adjustment | 279 | 46 | 0.58 (0.43-0.80) | 1.88 (1.32-2.68) | 4.46 (2.05-9.71) | 2.49 (1.07-5.78) | 0.35 (0.16-0.76) | 0.49 (0.23-1.04) |
| Strict analysis set: excluding non-depletion-related early discontinuation and unexplained low-consumption records | 285 | 55 | 0.55 (0.40-0.74) | 1.82 (1.32-2.51) | 3.76 (1.81-7.81) | 2.85 (1.26-6.46) | 0.47 (0.24-0.93) | 0.48 (0.24-0.97) |

Values are adjusted odds ratios (ORs) with 95% confidence intervals for the persistent high-demand phenotype. All models included age, surgical duration, open surgery versus laparoscopy, hysterectomy performed, regional block, and intraoperative dexmedetomidine. Age and surgical duration were scaled per 10-year and 60-min increases, respectively. Reference categories were laparoscopy, no hysterectomy, no regional block, and no intraoperative dexmedetomidine. The phenotype thresholds derived from the overall cohort—cumulative 0–24 h consumption >19.6 mg and incremental 24–48 h consumption >18.2 mg—were retained across all sensitivity analyses.

Non-depletion-related early discontinuation included discontinuation because of adverse events, patient refusal, or a change in the analgesic strategy. Unexplained low-consumption records were defined as cumulative 48-h consumption <12 mg without documented early PCIA discontinuation or PCIA parameter adjustment. Ceiling-dose records were defined as cumulative 48-h consumption of 49 mg, indicating consumption near the 50-mg PCIA reservoir limit. The strict sensitivity set excluded patients with either non-depletion-related early discontinuation or unexplained low-consumption records. CI, confidence interval; OR, odds ratio; PCIA, patient-controlled intravenous analgesia.
